# Supplementary material for: An inertial sensor-based comprehensive analysis of manual wheelchair user mobility during daily life in people with SCI
Source: PLoS One. 2025 Sep 29;20(9):e0323050. doi: 10.1371/journal.pone.0323050 (PMC12478954; doi:10.1371/journal.pone.0323050)
Supplement: S1 Appendix — (PDF) [file pone.0323050.s003.pdf]

Appendix. Steps to synchronize wheelchair mounted sensors

Step 1: Calculate sensor orientation

Orientation quaternions were calculated from the raw accelerometer and gyroscope data using an open-source algorithm [28], the Mahony Attitude and Heading Reference System (AHRS) filter, designed for efficient inertial motion tracking. The algorithm employs a quaternion-based gradient descent method that estimates orientation by minimizing the error between measured and expected acceleration and magnetic field directions. This method enables accurate orientation estimation by correcting gyroscope drift using accelerometer and magnetometer data. For this study, magnetometer data were not used. The algorithm relied on accelerometer and gyroscope signals to compute the quaternion orientation for each sensor across the recording period. The AHRS filter was initialized with a sampling frequency of 100 Hz, corresponding to a sample period of 0.01 seconds. Raw tri-axial acceleration (in m/s^2) and angular velocity (in rad/s) data from the IMU were used as inputs to the filter without additional preprocessing. At each time point, the Mahony filter was updated using these raw signals, and the resulting orientation was stored as a quaternion $q = [q_0\ q_1\ q_2\ q_3]$, where q_0 is the scalar component and $[q_1\ q_2\ q_3]$ represent the vector component.

This step provided a continuous estimate of sensor orientation over time. The resulting quaternion time series was subsequently used to transform raw sensor measurements from the sensor-fixed frame to a global (inertial, world-fixed) reference frame.

Step 2: Resolve sensor measurements into the inertial (world) frame

To resolve angular velocity measurements from the sensor-fixed frame to the inertial (world) frame, quaternion-based rotation was employed. At each time point, the sensor orientation was represented as a unit quaternion.

Sensor-Specific Considerations:

- **Chair-mounted sensor:** Maintained consistent orientation relative to the wheelchair frame throughout data collection
- **Wheel-mounted sensors:** These sensors rotate continuously around the wheel axis during wheelchair pivot maneuver. The quaternion-based transformation accounts for this rotation, allowing extraction of motion components in the world-fixed frame despite the sensor's changing orientation relative to the wheelchair

The transformation of a vector w into the world frame was performed using the quaternion rotation formula:

$$\boldsymbol{w_{world}} = \boldsymbol{q} \otimes \boldsymbol{w_q} \otimes \boldsymbol{q}^* \tag{1}$$

Here, $\otimes$ denotes the quaternion product, q^*, is the quaternion conjugate of q, and $w_q = [0\ w_1\ w_2\ w_3]$ is the vector w expressed in quaternion format with a zero-scalar part. The rotated vector is then extracted from the vector part of the resulting quaternion.

Quaternion Conjugate:

$$\boldsymbol{q^*} = [\boldsymbol{q_0} - \boldsymbol{q_1} - \boldsymbol{q_2} - \boldsymbol{q_3}] \tag{2}$$

Quaternion Product:

Given two quaternions $a = [a_0\ a_1\ a_2\ a_3]$, and $b = [b_0\ b_1\ b_2\ b_3]$, their product $a \otimes b$ is computed as:

$$\begin{aligned} (\boldsymbol{a} \otimes \boldsymbol{b})_0 &= \boldsymbol{a_0 b_0} - \boldsymbol{a_1 b_1} - \boldsymbol{a_2 b_2} - \boldsymbol{a_3 b_3} \\ (\boldsymbol{a} \otimes \boldsymbol{b})_1 &= \boldsymbol{a_0 b_1} + \boldsymbol{a_1 b_0} + \boldsymbol{a_2 b_3} - \boldsymbol{a_3 b_2} \\ (\boldsymbol{a} \otimes \boldsymbol{b})_2 &= \boldsymbol{a_0 b_2} - \boldsymbol{a_1 b_3} + \boldsymbol{a_2 b_0} + \boldsymbol{a_3 b_1} \\ (\boldsymbol{a} \otimes \boldsymbol{b})_3 &= \boldsymbol{a_0 b_3} + \boldsymbol{a_1 b_2} - \boldsymbol{a_2 b_1} + \boldsymbol{a_3 b_0} \end{aligned} \tag{3}$$

Step 3: Filter angular velocity in the inertial frame

To reduce high-frequency noise in the angular velocity signal, a low-pass Butterworth filter was applied to the z-axis component of the angular velocity measured in the world-fixed reference frame. A fourth-order filter was implemented with a cutoff frequency of 0.5 Hz. The filtering process was applied using zero-phase forward and reverse filtering to prevent phase distortion in the signal. The normalized cutoff frequency was computed relative to the Nyquist frequency (half the sampling rate). The resulting filter coefficients were then used to process the z-axis angular velocity component $w_{world_z}(t)$ yielding a smoothed signal $w_{world_{z,filt}}(t)$.

Step 4: Sensor synchronization

In this study, data from the wheelchair-mounted inertial measurement units (IMUs) were used to analyze movement. To enable consistent temporal alignment across sensors, a manual synchronization procedure was implemented using filtered angular velocity signals in the inertial frame ($w_{word_{z,filt}}$) from each sensor. This approach ensured a reliable comparison of motion data across sensor locations, particularly during turning maneuvers (Fig 1).

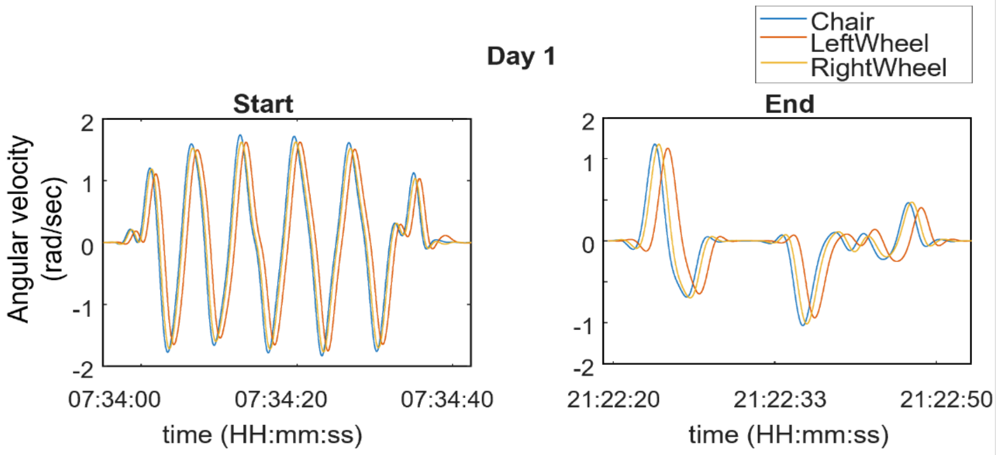

Figure 1. Angular velocity in the z-axis (yaw rotation) measured from three IMU sensors for one representative participant on Day 1: Left figure shows data from the start of the day displaying wheelchair pivot maneuver. Right panel shows wheelchair maneuver data from the end of the day.

Synchronization procedure

To align all sensors in time, wheelchair pivot maneuvers were selected as common synchronization landmarks due to their distinctive angular velocity signatures that could be reliably detected across all sensor locations. The synchronization procedure involved the following steps:

1. **Turn Event Selection:** Two prominent pivot maneuvers were identified at the beginning and end of each recording day.
2. **Cross-correlation Analysis:** Time lags between the angular velocity signals from each sensor and the reference sensor (chair-mounted) were calculated using cross-correlation.
3. **Drift Estimation:** Linear drift rates were computed based on the time lag differences between the two synchronization events.
4. **Drift Correction:** A linear drift correction was applied across the entire recording period between the two selected turns.
5. **Signal Resampling:** Angular velocity signals for each sensor were resampled using linear interpolation to match the time base of the reference sensor.

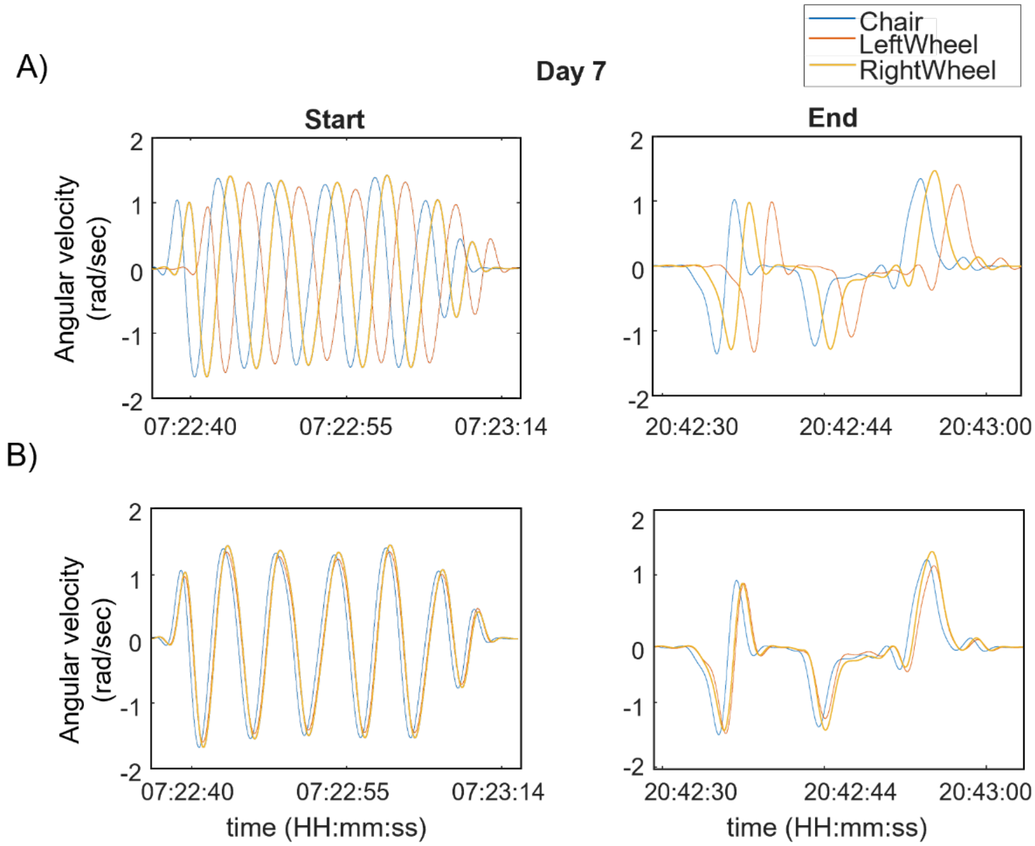

Figure 2. Angular velocity in the z-axis (yaw rotation) measured from three IMU sensors for one representative participant on Day 7, showing data before and after synchronization. (A) demonstrates the raw, unsynchronized data where temporal misalignment between chair-mounted, left wheel, and right wheel sensors is evident. (B) shows the same data after applying the manual synchronization procedure, resulting in improved temporal alignment across all three sensor locations, enabling reliable comparison of motion data during turning maneuvers.

Synchronization performance and drift analysis

Daily Synchronization Frequency: Given the extended recording period and long periods of data used for analysis (up to 12 hours per day), we performed a clock drift analysis to evaluate whether once-daily synchronization was sufficient. Temporal drift analysis revealed that clock synchronization degraded progressively over extended recording periods, with maximum observed drift between sensors reaching several seconds by the end of the recording period (7 days).

Drift Characteristics: The drift patterns were consistent across recording days but varied between sensor locations. As shown in Figure 3 for a sample data trial, the left wheel sensor exhibited the most pronounced drift relative to the chair sensor (>3 seconds by Day 7), while the right wheel sensor showed more gradual drift accumulation (~1 second by Day 7). While the data shown in Figure 3 is representative of one participant, all other participants exhibited similar drift behavior patterns across their respective sensor configurations.

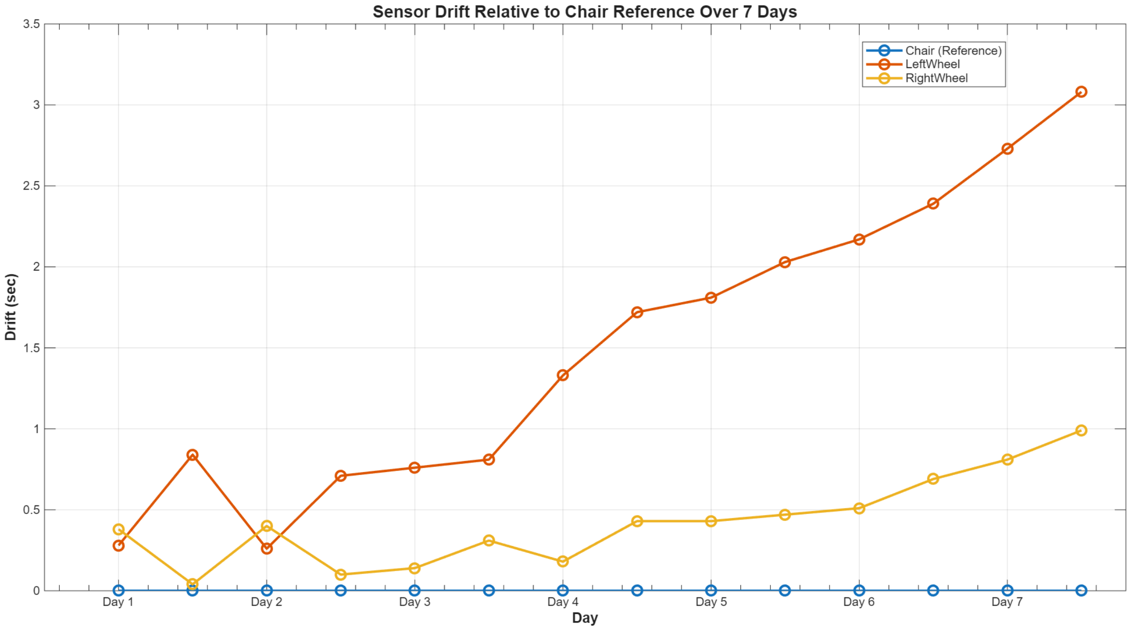

Figure 3. Sensor drift relative to chair reference over seven days.

Correction Range: Synchronization corrections typically ranged from tens of milliseconds in the morning to several seconds by the end of the recording day. The linear interpolation approach provided adequate correction for the observed drift patterns, maintaining temporal alignment accuracy within ±50 milliseconds across all sensors after synchronization.

Multi-day Considerations: For the seven-day monitoring period, daily synchronization was deemed sufficient given the linear nature of the observed drift. However, for longer recording periods or applications requiring higher temporal precision, more frequent synchronization events would be recommended.
