# Supplementary material for: An inertial sensor-based comprehensive analysis of manual wheelchair user mobility during daily life in people with SCI
Source: PLoS One. 2025 Sep 29;20(9):e0323050. doi: 10.1371/journal.pone.0323050 (PMC12478954; doi:10.1371/journal.pone.0323050)
Supplement: Attachment — Submitted filename: Response to Reviewers.pdf [file pone.0323050.s005.pdf]

**PLOS ONE PONE-D-25-16850 Response to Reviewers**

**We thank the reviewers for their detailed and helpful feedback. We diligently reviewed the critiques, questions, comments, and suggestions and responded as thoroughly as possible. Our responses are detailed below; reviewer comments are presented in normal text, our responses are presented in bold text, and relevant edits to the manuscript are presented in bold italics.**

<u>Reviewer #1:</u> An inertial sensor-based comprehensive analysis of manual wheelchair user mobility during daily life in people with SCI

*General:*

- Carefully written manuscript on wheelchair user mobility monitoring with IMUs.

- Reference 10 is not on wheelchair mobility but shoulder load, probably a mix-up with another publication of the author on wheelchair mobility metrics

  <u>**Authors' response:**</u> **The previous reference 10 was replaced with the correct reference (now reference 17). Thank you for catching our error!**

  ***"de Vries WHK, van der Slikke RMA, van Dijk MP, Arnet U. Real-Life Wheelchair Mobility Metrics from IMUs. Sensors. 2023;23: 7174. doi:10.3390/s23167174 "***

*Methods*<u>**:**</u>

- **line 109:** when data is not used, there is no need to describe the sensors on the wheelchair user ... saves you some words

  <u>**Authors' response:**</u>

  **Following the Reviewer's suggestion, the description of the sensors that were not used for this analysis were removed. *"...We utilized three IMUs (Axivity-AX6; range ±16 g and ±2000 deg/s, sampling frequency 100 Hz, weight 11g) that were placed on the MWC (frame and wheels) (Fig 1). "***

- **line 111:** recoding? or recording ?

  <u>**Authors' response:**</u>

  **Thank you for catching our error. The word is supposed to be 'recording'. The sentence has been corrected: *"... The IMUs used in this study allowed seven days of continuous data recording without recharging when operating in full IMU mode (recoding both linear acceleration and angular velocity) recording at 100 Hz."***

- **line 132:** I do understand wat you do for synchronization, but your description is far from complete. First, you should describe how your sensor is aligned with gravity, which is per definition impossible for the wheelsensor, as it will turn around the wheelaxis during the pivoting

movements. Just mentioning "a vertical axis" is not sufficient here. Furthermore, a vertical axis refers to a real world coordinate system, where inertial reference frame refers to a local (sensor) reference frame. Please be as concise as possible.

**Authors' response: We created a supplementary document to explain how synchronization was performed (Supplement 3) to provide more information to the reader and address some questions from the other reviewer.**

***"S3 Appendix. Steps to synchronize wheelchair mounted sensors."***

- **line 138:** please describe in more detail how you calculate orientation quaternions from angular velocity and accelerations.

  **Authors' response: To estimate the orientation of the inertial measurement unit (IMU) over time, an open-source implementation of the Mahony Attitude and Heading Reference System (AHRS) filter was used [28]. The MahonyAHRS algorithm implementation used here is open-source and available in GitHub. [29]. Given the comprehensive documentation provided in the referenced literature and the GitHub repository, we chose not to repeat these implementation details in this manuscript. However, we did update the manuscript to ensure it is clear where the interested reader can find the relevant documentation.**

  **The description in the manuscript was also updated and is copied under the next comment.**

  ***"Orientation quaternions were calculated from the raw accelerometer and gyroscope data using an open-source algorithm [28,29] designed for efficient inertial motion tracking. The algorithm employs a quaternion-based gradient descent method that estimates orientation by minimizing the error between measured and expected acceleration and magnetic field directions. This method enables accurate orientation estimation by correcting gyroscope drift using accelerometer and magnetometer data. For this study, magnetometer data were not used. The algorithm relied on accelerometer and gyroscope signals to compute the quaternion orientation for each sensor across the recording period."***

- Furthermore, at **line 148-149** you suddenly talk about direction cosine matrix. please be consistent in your wording.

  **Authors' response: We have addressed the inconsistency in terminology by adding a transitional sentence that connects the quaternion calculations to the direction cosine matrix discussion. The revised text now includes a clear explanation of why quaternions are converted to direction cosine matrices, providing better continuity between these concepts. The added sentence reads:**

  ***"These quaternions were subsequently converted to direction cosine matrices to facilitate coordinate frame transformations [30]. The direction cosine matrix that defines the***

***transformation of measurements made in the sensor-fixed frame to those in the inertial frame was defined as $R_{inertial|IMU}$."***

- **line 152-153:** nice approach :-)!!
  **<u>Authors' response:</u> Thank you!**

- **line 157-158:** hope you multiplied by 2*PI as well ??? A simple formula would be helpful here to remove any doubt. Was any filtering applied before numerically differentiating into wheelchair linear acceleration??
  **<u>Authors' response:</u>**
  **We appreciate the reviewer's attention to detail. Yes, the conversion from angular velocity to linear speed was handled by first computing the wheel radius as r = circumference / (2π) and then using the relationship v = r * ω to compute the linear speed of the wheelchair from the wheel's angular velocity (in rad/s). The angular velocity values were already in radians per second, so additional multiplication by 2π was not necessary. Linear acceleration was then calculated by numerically differentiating the estimated speed using MATLAB's gradient function. No filtering was applied prior to the differentiation step.**

  **The following sentences were edited:**

  ***"The linear velocity of each wheel was calculated by multiplying the wheel rotation velocity (in rad/s) by the wheel radius, which was derived from the measured wheel circumference. Wheelchair speed ($v_{WC}$) was the average of the calculated linear velocities of both wheels. Wheelchair linear acceleration ($a_{WC}$) was obtained by numerically differentiating wheelchair speed. No filtering was applied prior to differentiation."***

- **line 169-171:** you also assume or checked/forced the wheelchair is standing on level ground for this operation?
  **<u>Authors' response:</u> Yes, we assumed that the wheelchair was on level ground for this operation. This assumption was verified in two ways: first, through visual inspection of the wheelchair's position at the beginning of each day, and second, by comparing the calculated acceleration of the chair with the acceleration measured by the wheelchair sensor. This comparison ensured consistency and confirmed that the wheelchair was indeed stationery and on level ground during the relevant portions of the data.**

  **The following sentences were edited:**

  ***"The average acceleration due to gravity measured during no movement ($\vec{a}_{no\ movement}$) was used to define a wheelchair-fixed z-axis ($\hat{Z}_{WC}$) assuming that the axis is aligned with gravity [31] and the wheelchair was standing on level ground:"***

- **line 172:** you controlled that by placing and tightening the Frame sensor on the bar that connects to both wheel axes, which is per definition, by design, perpendicular wit with frontal axis.
  <u>Authors' response:</u> **Yes, the sensor was deliberately placed perpendicular to the frontal axis on the axle connecting bar.**
  **The revised manuscript now explicitly mentions that both the figures AND equations were updated to maintain consistency with the right-handed coordinate system. The equations presented in the paper are derived based on the right-handed coordinate system convention. Our implementation accounts for coordinate system orientation by computing rotations and transformations accordingly to ensure consistent results regardless of the initial orientation of the sensor coordinate frame. The code includes proper handling of these coordinate transformations to maintain the correct geometric relationships.**

  ***"We defined the forward direction axis ($X_{WC}$) by assuming that one sensor axis is already aligned well with a wheelchair axis through proper sensor mounting (Fig. 1). Given this mechanical alignment, the forward direction axis is calculated as:***

$$\hat{\mathbf{X}}_{\mathbf{WC}} = \frac{[\mathbf{1\ 0\ 0}] \times \hat{\mathbf{Z}}_{\mathbf{WC}}}{\mathbf{norm}\left([\mathbf{1\ 0\ 0}] \times \hat{\mathbf{Z}}_{\mathbf{WC}}\right)}$$

  ***And finally, we ensured that the $\hat{\mathbf{Y}}_{\mathbf{WC}}$ axis is orthogonal to the $\hat{\mathbf{Z}}_{\mathbf{grav}}$ and wheelchair axes:***

$$\hat{\mathbf{Y}}_{\mathbf{WC}} = \frac{\hat{\mathbf{Z}}_{\mathbf{gWC}} \times \hat{\mathbf{X}}_{\mathbf{WC}}}{\mathbf{norm}(\hat{\mathbf{Z}}_{\mathbf{WC}} \times \hat{\mathbf{X}}_{\mathbf{WC}})}$$

  ***The resulting unit vectors ($\hat{X}_{WC}, \hat{Y}_{WC}, \hat{Z}_{WC}$) define a wheelchair-fixed frame aligned to the wheelchair frame axes."***

- Figure 4. A is a right-handed coordinate system, B and C are left handed coordinate systems. Although for the metrics derived it doesn't matter too much (left and right turns might be exchanged, and upward or downward slopes), you should adapt your algorithms to it, or how you corrected for this, or describe how it could influence your outcomes (as you don't show any awareness of the difference currently)
  <u>Authors' response:</u> **Thank you for pointing this out. You are correct that Figure 4B and 4C showed left-handed coordinate systems, which did not match our approach. These have been corrected in the revised figure to match the right-handed coordinate systems used throughout our analysis.**

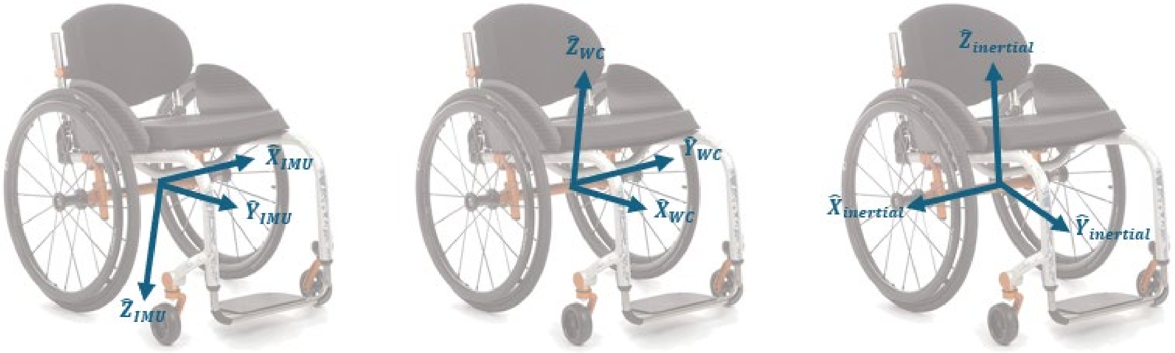


- **line 186:** why account for individuals who remain active past midnight. That activity just belongs to the next day. Otherwise, you should adapt your manuscript for wording, and talk about active period in between sleep periods, which sound quite silly.
  **<u>Authors' response:</u> Thank you for your feedback. We chose to define the 24-hour period from 3:00 AM to 2:59 AM the following day to accommodate participants whose activity patterns do not align with a traditional daytime schedule; for example, individuals who work night shifts or have irregular rest patterns. This approach ensures that all loading within a natural activity-rest cycle is captured in a single analysis period, rather than arbitrarily splitting it across calendar days. While we understand the suggestion to use traditional day boundaries, doing so could artificially divide meaningful bouts of arm use and recovery, which may misrepresent the true daily load on the upper extremities. We clarify our method in the sentences below.**

  ***"…This choice was made to account for individuals who remain active past midnight, such as those with nontraditional schedules or night shifts. This approach prevents splitting periods of continuous activity and provides a more accurate representation of daily arm loading."***

- **line 189 – 195:** any references for these cutoff numbers or just decided in discussion based on pilot data analysis? this phrasing is still not informative on how you came to these numbers. Any graphs supporting the choice would be beneficial.
  **<u>Authors' response:</u> There is a current lack of standardized definitions for wheelchair turning events in real-life settings [10]. Therefore, we determined our threshold values through systematic testing on controlled movement patterns and validation on both pilot data and real-world data collected in this study, similar to the approach of [14]. While these thresholds require further validation across different populations and environments, they provide a consistent framework for turn detection that can be refined through future research.**

**The figure below shows the angular velocity and the trajectory of the wheelchair where green represents the start of the turn and red represents the end of the turn, demonstrating the effectiveness of our threshold-based approach in identifying meaningful turning events in real-world conditions.**

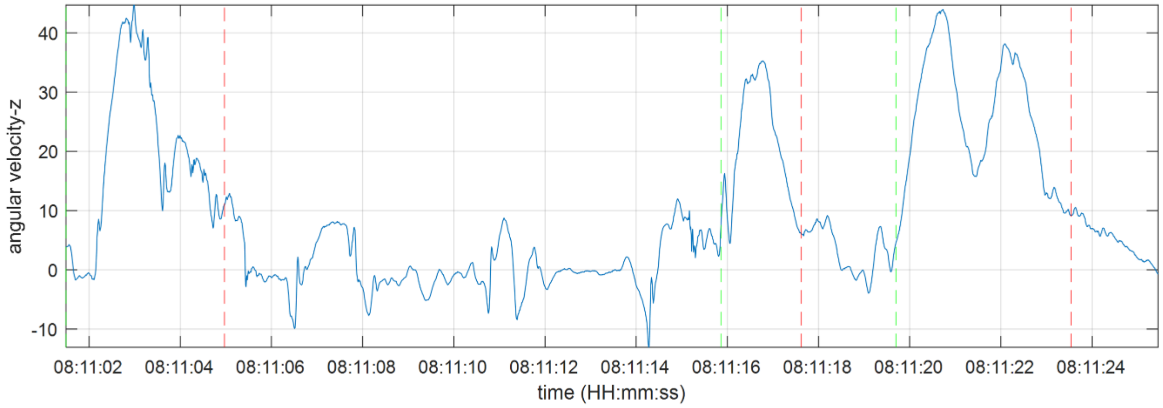


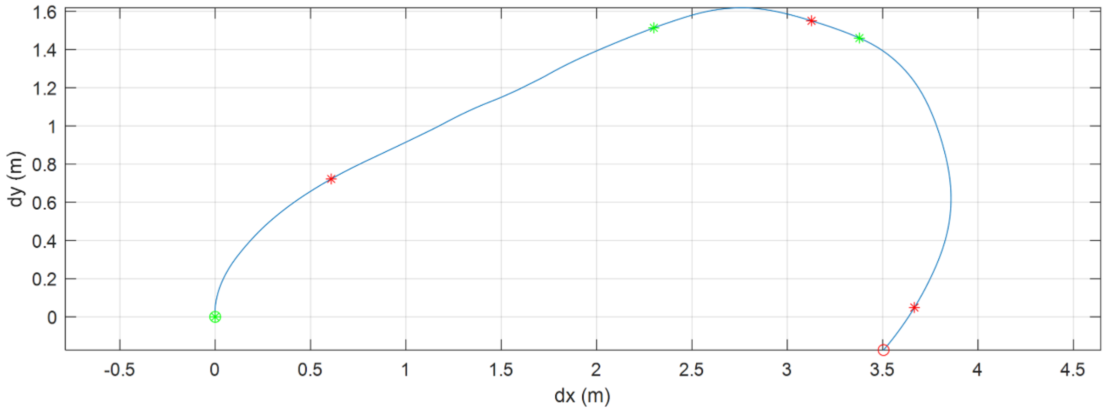


- **table 2:** Slopes. Good one that you take slope during a mobility bout. This is the way to distinguish between slopes, and just leaning backward "at rest". Please discuss it shortly, as many readers might not be aware of the distinction, when applying these methods.
  **Authors' response: We added a description to definition to clarify these methos:**

  ***"Average incline or decline encountered during a mobility bout. This measure helps distinguish between actual slope navigation during movement and simply leaning backward or forward while at rest."***

- **line 213:** I don't get this. displacement (change in position) is calculated from yaw angle and wheelchair velocity??? Why not just numerical integration of linear velocity? do you expect the wheelchair to move sidewards? Is it the orientation of the inertial frame with respect to the wheelchair, or the wheelchair wrt to the inertial frame (as the latter, the inertial frame, is global)
  **Authors' response:**

**We use yaw angle decomposition rather than direct velocity integration for two key reasons: first, the wheelchair's linear velocity is measured along its forward axis, which changes direction as it turns, so we need to project this into global x-y coordinates based on its instantaneous orientation; second, direct numerical integration of linear velocity can introduce cumulative drift errors over time. Our method decomposes the forward speed into global x and y components using the yaw angle, then integrates these components to calculate accurate displacement in the wheelchair-fixed frame with respect to the inertial frame.**

- **line 223:** so negotiating slopes with a duration of shorter than 5 seconds could be missed. Please elaborate on this in the discussion.
  **<u>Authors' response:</u> Yes, this is indeed a limitation of our methodology. Slopes with durations shorter than 5 seconds could potentially be missed due to our filtering approach. However, since we are calculating the average slope over the duration of each mobility bout, our filtering approach does not impact our overall analysis.**

  **Additionally, we implemented a moving mean filter to smooth the collected data, as the pavement surface generates considerable vibrations that can introduce noise into the slope measurements. This filtering approach helps reduce the impact of surface irregularities and vibration-induced artifacts, though it does contribute to our inability to capture very short-duration slope changes. While this represents a limitation in terms of detecting brief slope variations, it aligns with our research objective of characterizing the overall slope conditions during mobility bouts rather than analyzing instantaneous slope fluctuations. We added the limitation to our manuscript.**

  ***"…Our methodology may miss slopes lasting less than 5 seconds due to the use of a moving mean filter to reduce vibration-induced noise. While this could prevent detection of brief steep inclines or declines, it has minimal to no impact on our analysis since we calculate average slope per mobility bout, which smooths short-term fluctuations and reflects overall incline conditions."***

- **line 231:** Why did you use a k-means cluster analysis, is a simple histogram not enough. How many groups were chosen for the clustering?
  **<u>Authors' response:</u> We used k-means cluster analysis rather than simple histograms to better capture natural groupings and patterns within the mobility bout data across multiple metrics simultaneously (duration, continuity, mean speed, total distance, and displacement). Unlike histograms, which show the distribution of a single variable at a time, k-means clustering allows us to classify bouts based on the combined characteristics of these features, providing a more comprehensive understanding of different mobility bout types.**

**The number of clusters was determined using the elbow method, which balances model complexity and explained variance. Based on this method, we selected 3 clusters as the optimal grouping, allowing meaningful interpretation of distinct mobility bout categories in the data.**

***"...A k-means cluster analysis was performed to categorize mobility bouts. This method enabled grouping bouts with similar movement characteristics across multiple dimensions, revealing distinct patterns of mobility. The number of clusters was chosen using the elbow method [34], which determined that 3 clusters was the ideal number."***

- **line 238:** And? was there any statistical difference between groups defined?
  **Authors' response:** **K-means clustering itself is an unsupervised learning method designed to partition data into groups based on feature similarity; it does not directly test statistical differences between groups. Instead, it identifies clusters by minimizing within-group variance. In this exploratory analysis, the primary goal was to identify natural groupings of mobility bouts based on multiple combined features rather than to test specific hypotheses about group differences. Because the clusters reflect data-driven groupings rather than experimental conditions, formal statistical comparison between clusters was not conducted. Instead, the focus was on describing the characteristics of each cluster to inform further targeted analyses in future studies.**

  ***"...Given the exploratory nature of this analysis, clusters were identified to describe natural groupings within the data, without conducting formal statistical comparisons between groups."***

**Discussion:**

- Table 6: Why this selection of studies for comparison? As there are many more, with the same metrics!!
  **Authors' response:** **We selected these studies for comparison because they collected data over extended periods, allowing for a more comprehensive assessment of mobility patterns. Additionally, the participant characteristics in these studies were similar to ours in terms of demographics and clinical profiles, which enhances the relevance and validity of the comparisons.**

  ***"...Table 6 compares our findings to the results of five other studies [11,12,14–16] on MWC mobility in adults. These studies were chosen for comparison because they similarly focused on manual wheelchair mobility in adults with SCI, used comparable metrics, and collected data over extended durations, making their findings relevant and comparable to ours."***

- **General question:** can you/did you distinguish manual wheelchair propulsion from any form of power assisted wheelchair ambulation? If not, how could any unmarked power assisted wheelchair ambulation influence your results?
  **Authors' response:** **In this study, participants did not use any form of power-assisted wheelchair ambulation; all mobility bouts analyzed were from manual wheelchair propulsion. Therefore, the data represents solely manual wheelchair use. If power-assisted ambulation had occurred but was unmarked, it could potentially affect the measured mobility metrics—such as increasing distance traveled at higher speeds—which might alter the mobility profile for that individual. However, since our participants did not use power-assisted devices, the results accurately reflect their typical daily manual wheelchair routines.**

- **line 414:** Do you have plans to develop methodology to identify surface type from IMU data? Should be partly feasible, when analyzing wheelchair frame sensor data, as higher frequency oscillations on the vertical axis might correlate with pavement type ...
  **Authors' response:** **Yes, we have plans to develop methodology to identify surface type from IMU data, and we are currently working on this approach. We agree that this is a promising direction, particularly given that higher frequency oscillations on the vertical axis can correlate with different pavement types and surface characteristics.**

  **Our current work is expanding beyond the wheelchair frame sensor to utilize data from all three IMU locations (wheelchair frame, wheels and wrist) to develop a comprehensive surface classification system. The multi-sensor approach allows us to capture different aspects of surface-related vibrations and movements - the frame sensor captures direct wheelchair-surface interactions, while the body-worn sensors provide information about how surface characteristics translate into user movement patterns**

- And then, the final big question: How to relate all this detailed information to shoulder problems?
- And: is the load of wheelchair ambulation the one and only factor that influences progress of shoulder problems (pain, pathology) in manual wheelchair users???
  **Authors' response:** **We thank the Reviewer for this comment. A paragraph to highlight this question was added to the Discussion.**

  ***"Relating detailed mobility and biomechanical data to shoulder pathology in manual wheelchair users is complex, as shoulder problems likely result from a combination of factors. While load from wheelchair use is an important contributor, it is only one piece of the puzzle [39]. Other key contributors include repetitive loading, propulsion technique, transfer mechanics, seating posture, arm use during activities of daily living, injury history, muscle strength and conditioning, as well as individual anatomical, health, and psychosocial factors that influence pain perception and behavior. Our high-resolution mobility data provides objective quantification of one key component (wheelchair***

***propulsion exposure) but must be integrated with clinical assessments of these other factors to develop a comprehensive understanding of shoulder pathology risk. Future studies should combine our mobility analysis approach with longitudinal clinical evaluations, imaging, and patient-reported outcomes to establish how mobility patterns interact with individual risk factors to influence shoulder health outcomes. Our data on mobility bouts and biomechanical loading provides valuable insight, but integrating these findings with clinical assessments and longitudinal monitoring of shoulder health is essential for understanding the multifactorial nature of shoulder pathology and informing strategies to prevent or mitigate it."***

---

<u>Reviewer #2:</u>

Review notes

- This study is a valuable addition to gaining insight into the mobility profiles of wheelchair users. With increasing emphasis on the importance of physical activity, information about activity in wheelchair users is still lagging. So, compliments for taking on this challenge. Although the numbers are not that large yet, this is a good first step toward gaining more insight.

<u>**Abstract**</u>

- The abstract is clear but could be more powerful if two points are added. Can you include information about the reliability/accuracy of the method used? Also, some numbers about activity are provided, but how do they relate to previous research? Or, if such comparisons are not available, how do they compare to the able-bodied population (e.g., active time)?

  <u>**Authors' response:**</u> **Thank you for this suggestion. We did not do any specific reliability or accuracy testing of our method in this study, but, given that our approach is physics-based, we have no concerns about the reliability or accuracy of our calculated measures. While we did make comparisons to other studies of MWC users, we feel that comparisons to the able-bodied population are outside of the scope of this study. Our study is focused on presenting the approach and an analysis of a small sample. Therefore, we did not modify the abstract.**

<u>**Introduction**</u>

- Overall, it's comprehensive, well-cited, and logically structured. It provides a clear rationale for the study and situates it within relevant literature.
- There is a strong emphasis on both clinical relevance (e.g., secondary health risks) and technological innovation (e.g., IMUs). Prior work is well-referenced, and there is a clear progression from general context to specific gaps in knowledge.

  <u>**Authors' response:**</u> **We thank the reviewer for their supportive comments.**

- While "mobility profile" is introduced as a novel approach, it's not clearly defined. Consider clarifying how these profiles are generated or used (e.g., clustering? classification?).

  <u>**Authors' response:**</u> **We added a description of the term 'mobility profile':**

***"…Additionally, we incorporated a more detailed analysis of MWC maneuvering, including the number of turns, starts, and stops, into a novel approach that we call a mobility profile. A mobility profile provides a weekly summary that quantifies maneuvering behaviors, categorizes bout durations using data-driven thresholds (short <215s, medium 215s-700s, long >700s), and assesses environmental demands to provide comprehensive insight into movement patterns through cluster analysis."***

- **L54–77** This part could be condensed a bit; currently, there is some redundancy.
  Authors' response: **Thank you for this feedback. We agree that this section contained redundant information across multiple studies reporting similar mobility metrics. We have condensed this section by eliminating repetitive speed and distance measurements, grouping similar findings together, and highlighting key distinctions between different environments (e.g., residential vs. care facility settings) while maintaining all essential information.**

- **L80** Could you describe in a bit more detail what is meant by "different environmental contexts"? While it's clear to me, a more general audience might benefit from examples, and from an explanation of how detailed motion measurements might provide more insight.
  **Authors' response: We provide a more detailed description in the revised manuscript.**

  ***"Environmental contexts include diverse settings from indoor spaces (homes, offices, stores) to outdoor areas (sidewalks, parks, campuses), each with distinct challenges like different surfaces, obstacles, and crowds. Context-specific motion measurements could reveal how wheelchair users adapt their movement strategies across these varied environments, offering deeper insights than general mobility metrics."***

- **L82** The shift from discussing the literature to introducing the study could benefit from a clearer, explicit statement. Add something like: "To bridge this gap..."
  **Authors' response: Thank you, a sentence has been added per the reviewer's recommendation.**

  ***"To bridge this gap, our study built upon previous research by developing a comprehensive analysis of MWC mobility by employing data measured by IMUs to assess and quantify detailed characteristics of MWC use during daily life."***

Methods

- Participants: Consider briefly justifying the small sample — is this a pilot study, or a sub-analysis from a larger trial?
  **Authors' response: This represents a sub-analysis from a larger trial, for which data collection is still ongoing. The current sample was selected to conduct preliminary**

**analyses and inform the development and refinement of analytic methods prior to applying them across the full cohort. Details have been added to explain the small sample size:**

***"Real-world data was collected from a cohort of 12 MWC users with SCI (Table 1) over seven consecutive days as part of an observational, longitudinal study focused on wheelchair mobility and arm use in daily life."***

<u>Data collection</u>

- **L109** You state that sensors were placed on the thorax, upper arms, and forearms, but this data was not used. Why was this data collected but excluded from this analysis? If relevant to another study, a short note would clarify.
  **<u>Authors' response:</u> We appreciate the reviewer's observation. Sensors were placed on the thorax, upper arms, and forearms as part of the broader data collection protocol designed to support multiple analyses. While this study focused on wheelchair mobility outcomes, additional sensor data was collected for a separate analysis focused on arm use and upper extremity movement. To avoid confusion, we revised the manuscript to only describe the sensors relevant to this analysis.**

  ***"…We utilized three IMUs (Axivity-AX6; range ±16 g and ±2000 deg/s, sampling frequency 100 Hz, weight 11g) that were placed on the MWC (frame and wheels) (Fig 1). "***

- No mention is made of how data quality was checked — were any sensors dislodged? Was there missing data? Were any days excluded from analysis?
  **<u>Authors' response:</u> We thank the reviewer for this important point. Data quality was visually inspected using the Axivity software (OmGui) to ensure completeness and integrity. No days of data collection were missing or excluded due to sensor malfunction. All sensors remained securely attached throughout the recording period, and no dislodgement was observed. The only days excluded from the analysis were those exceeding the 7-day data collection window. We have added the following clarification to the manuscript:**

  ***"…Upon completion of the data collection period, a research team member retrieved each set of sensors during an in-person visit. After data download, data were visually inspected using the manufacturer's software to confirm signal quality and completeness. Days beyond the designated 7-day period were excluded from analysis."***

- **L119** A synchronization movement is used, but since synchronization for the remainder of the day relies on the internal clock of the devices, is once per day enough? Did you analyse drift or variation in sync over the course of a day, and were these consistent across days? If corrections were needed, in what range were they — milliseconds or minutes?
  **<u>Authors' response:</u> Thank you for your question regarding synchronization frequency and drift analysis. To address your concerns, we can reference the work by Galna et al. (2025),**

**who demonstrated that desynchronization between unconnected IMUs follows predictable patterns with corrections remaining in the millisecond range. Their findings show that once-per-session synchronization is sufficient, supporting the feasibility of reduced synchronization frequency while maintaining acceptable precision.**

**Supporting information was included to explain how synchronization was performed in our study and to provide some example data of the drift we observed between sensors.**

***"S3 Appendix. Steps to synchronize wheelchair mounted sensors."***

Galna, B., Wood, E., Griffiths, S., Jackson, D., Rivadella, A., & Spears, I. (2025). Synchronisation of multiple unconnected inertial measurement units using software correction. *Journal of Biomechanics*, 183, 112632.

**Mobility metrics**

- This is a clear section, well described. However, the settings and thresholds used are based on in-lab pilot measurements, which could affect their applicability to outdoor or real-world results. It would be good to evaluate how well these settings worked for the collected data.
  **Authors' response:** **The threshold settings were also tested and evaluated on the real-world outdoor data collected in this study. The figure below shows an example of a movement bout from our dataset, angular velocity over time, with distinct peaks marking the turning events, while the lower panel shows the corresponding spatial trajectory with the identified turning points marked along the path. This example illustrates that our thresholds effectively captured meaningful turning behavior in the outdoor environment, confirming the applicability of our parameter settings to real-world wheelchair navigation.**

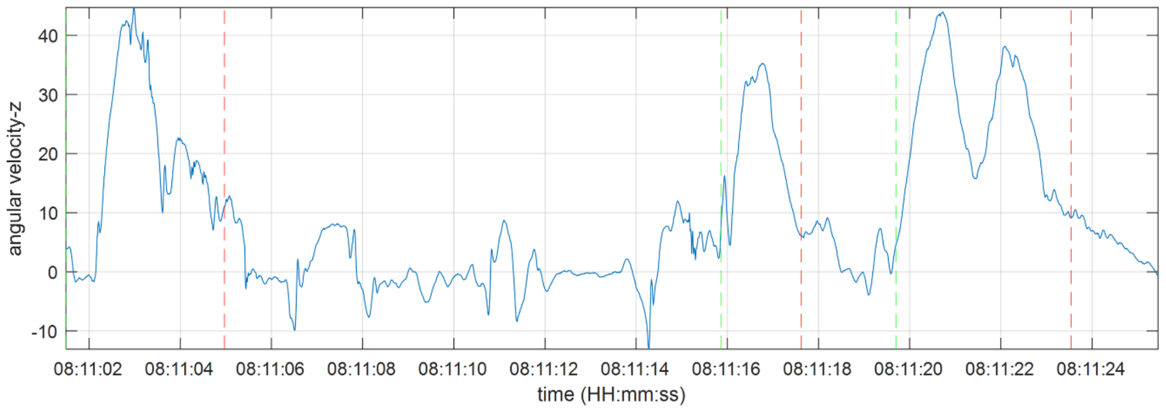


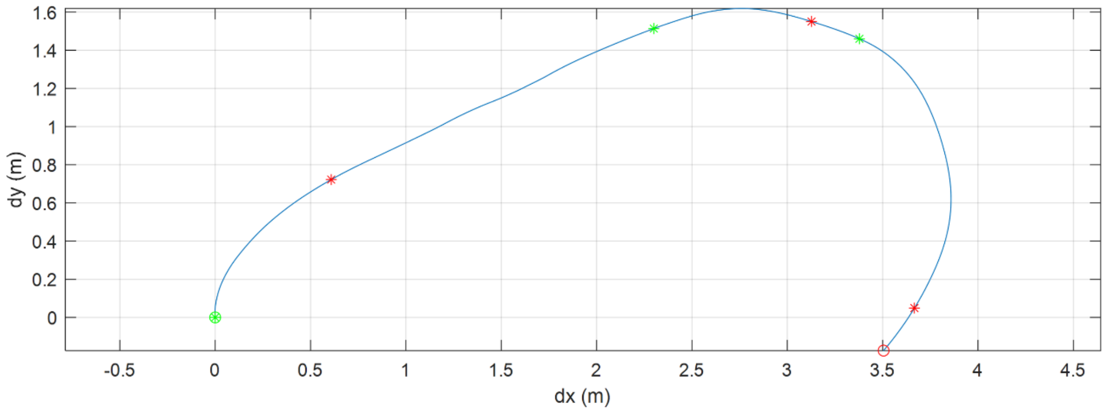


- Currently, it's stated that the settings are based on pilot studies, but this lacks rationale. Was this based on visual inspection, trial-and-error, or comparison to ground truth? For example, why was a 0.05s gap allowed for turning? Why not use a change in direction threshold instead?

  **Authors' response: We based our turn detection settings on visual inspection of both pilot data and trial data (see above). The suggestion of using a change in a direction threshold is interesting, but using only direction change would capture every minor course correction or navigation adjustment, resulting in an inflated count of insignificant movements that don't represent meaningful turning behavior. Our 10°/sec angular velocity threshold with 0.05-second pause tolerance specifically targets intentional turning maneuvers while filtering out minor directional adjustments, wheelchair vibrations, and small corrective movements that are inherent in real-world navigation but don't constitute purposeful turns. We did look into the sensitivity of turn detection to our pause threshold (data for 1 day for 1 participant shown below); as expected, a larger threshold results in less turns detected. However, the turn detection is not overly sensitive to this choice, as a 1000% increase in the pause tolerance (to 0.5-seconds) results in only an 8% drop in the number of turns detected.**

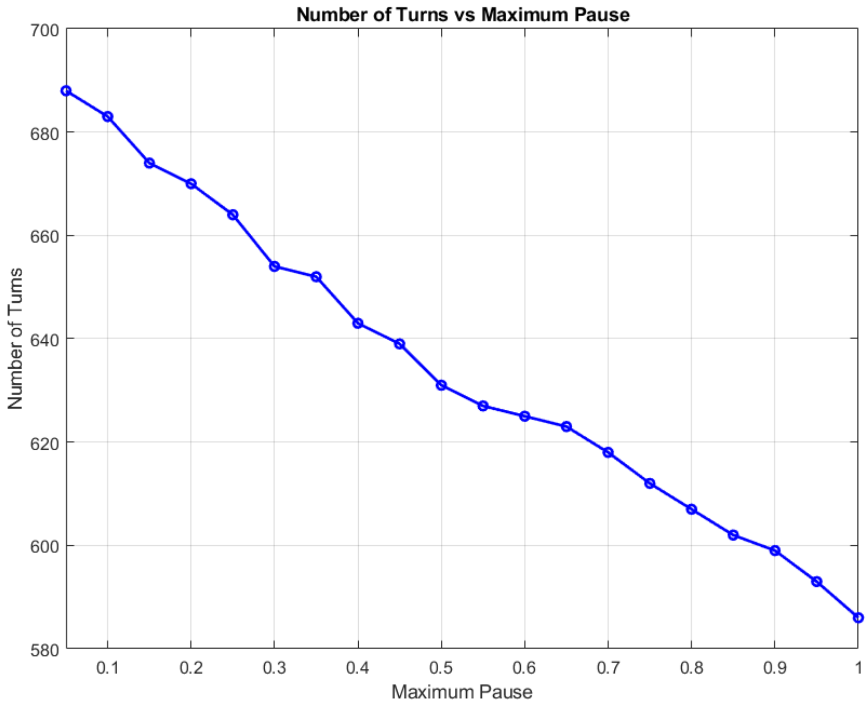


**<u>Statistical analysis</u>**

- It is stated that clustering was performed, but it's not explained how k (the number of clusters) was chosen.

  **<u>Authors' response:</u> Thank you for this important methodological question. We determined the number of clusters using the elbow method, a common approach for identifying the optimal number of clusters in k-means analysis. This method involves plotting the total within-cluster sum of squares against increasing values of k and identifying the point where the marginal gain in explained variance begins to diminish (the "elbow"). In our dataset, the elbow occurred at k = 3, which provided a balance between model simplicity and explanatory power.**

  **<u>The following sentence was added:</u>**

  ***"…The number of clusters was chosen using the elbow method [34], which determined that 3 clusters was the ideal number."***

- You mention "five metrics" (duration, continuity, mean speed, total distance, displacement), but it's unclear why these specific metrics were selected.

  **<u>Authors' response:</u>**

  **These five metrics (duration, continuity, mean speed, total distance, and displacement) were selected based on prior research examining wheelchair mobility using sensor-based approaches. Together, they capture both temporal and spatial aspects of movement and allow a multifaceted characterization of mobility bouts. Duration and continuity reflect the**

**temporal structure of movement; mean speed and total distance quantify the intensity and extent of mobility; and displacement reflects directional change and path efficiency. These metrics were chosen because they provide complementary insights into different aspects of wheelchair mobility that are relevant to real-world activity and are supported by prior literature.**

***"MWC mobility bouts were analyzed using five metrics (duration, continuity, mean speed, total distance, and displacement data, chosen based on prior studies of WC mobility and were selected to provide a comprehensive view of both temporal and spatial aspects of movement) from twelve participants. "***

- The examination of the data was done "visually" with "histograms and distribution patterns" — it would help to briefly state what specific patterns or anomalies you were looking for.
  **Authors' response: Visual examination using histograms and distribution plots was conducted to explore the range and variability of each mobility metric and to evaluate how well the identified clusters separated the data. Specifically, we looked for patterns such as multimodality (e.g., distinct peaks in bout durations or speeds), natural breaks or gaps in distributions, and consistency in cluster separation across participants. This step was used to assess whether the clustering solution aligned with interpretable and observable distinctions in the data, thereby supporting the use of cluster-informed thresholds in further analyses.**

**Mobility profiles**

- While the thresholds for bout duration were based on k-means, it's not clear how the numerical cutoffs (215s and 700s) were derived. Since the selection of bout durations directly affects the results and their interpretation, this is an important methodological choice that needs further explanation.
  **Authors' response: The numerical thresholds for bout duration (215s and 700s) were derived by visually inspecting the distribution of bout durations within the three clusters identified by k-means across all participants. We overlaid cluster labels onto the bout duration histograms and identified the approximate boundary points where transitions between clusters occurred. These thresholds were chosen because they consistently marked shifts in bout characteristics (e.g., from short to intermediate, and intermediate to long duration) across the sample. While approximate, this data-informed approach ensured that the thresholds reflected meaningful differences in bout patterns as revealed by the clustering results.**

  **The description has been updated:**

***"…Bout duration thresholds (215 s and 700 s) were derived by examining where natural breaks occurred between clusters across participants, allowing for classification of short, intermediate, and long-duration bouts based on real patterns observed in the data…. "***

**<u>Results</u>**

- Consider using some subheadings for a clearer structure. Currently, a lot of different analyses are presented together, making it dense to read.
- **<u>Authors' response:</u> Subheadings were added to the results section *"Mobility bouts" and "Mobility profile"***

- The term "continuity" is used repeatedly (e.g., "short bouts had high continuity"), but it's never explicitly defined.
  **<u>Authors' response:</u> Thank you for pointing this out. The term continuity was defined in Table 2 of the Methods section as:**
  ***"Ratio of time spent moving to the total duration of a mobility bout."***

  **This metric reflects how continuous movement is within each bout and was introduced as a novel measure for wheelchair mobility in our study, which parallels the continuity of a walking period used in [23].**

  **To improve clarity for readers, we have now explicitly restated this definition in the Results section when continuity is first mentioned, ensuring the term is clearly understood throughout the manuscript. *"… For example, short bouts had high continuity (87%; defined as the ratio of time spent moving to the total duration of a mobility bout), covered a mean displacement of 5.91 m…"***

**<u>Discussion</u>**

- I am not too enthusiastic about placing Table 6 in the discussion. Usually, this kind of comparison with previous work would belong in the introduction, to show existing gaps and how the new research addresses them.
  **<u>Authors' response:</u> We appreciate the reviewer's feedback regarding the placement of Table 6; we debated the placement of Table 6, and because it contains a comparison of our results to other studies, we settled on placing the table in the discussion. While we understand that literature comparisons are often presented in the introduction, we believe Table 6 serves a different purpose in our discussion section. Rather than identifying research gaps (which we addressed in the introduction), Table 6 specifically compares our empirical results with findings from previous studies to contextualize our outcomes and highlight the contributions of our work.**

  **This results-focused comparison is most meaningful after readers have been presented with our methodology and findings. Additionally, we have structured Table 6 to prioritize our**

**results by placing them at the top, making our study's contributions immediately apparent while providing the necessary comparative context.**

**We believe this placement enhances the discussion by allowing readers to evaluate our results against the existing literature with full knowledge of our study's design and outcomes. However, if the reviewer feels strongly about this placement, we can consider alternative organizational approaches.**

- Emphasize more clearly which specific metrics or analyses are novel (e.g., frequency of starts/stops, ADA slope violations), how they enhance existing methods, and what added insights they provide beyond traditional measures like speed and distance.
  <u>**Authors' response:**</u> **Thank you for this valuable feedback. We revised the manuscript to more clearly emphasize our novel contributions.**

  ***"…Our integrative approach to mobility profiling represents a novel contribution to wheelchair research by combining traditional measures (speed, distance, and mobility bouts) with previously unexamined metrics including start/stop frequency and ADA slope violations. These high-resolution mobility metrics provide critical context to conventional measures, revealing the physical demands and environmental challenges that measures of speed and distance alone cannot capture. The quantification of ADA slope violations is novel in wheelchair mobility research and offers insights into environmental barriers encountered during daily life… "***

- The explanation of differences between studies (e.g., athlete vs. non-athlete, different countries) is plausible but speculative and underdeveloped. Either strengthen your arguments with supporting evidence or acknowledge the uncertainty more clearly.
  <u>**Authors' response:**</u> **We thank the reviewer for the helpful feedback. We have edited the manuscript to better acknowledge the uncertainty:**

  ***"…Despite differences in study populations, daily distance outcomes are generally similar, except for one study on competitive athletes [15] and another conducted in a different country [14]. These discrepancies likely stem from multiple factors, though the relative contribution of each cannot be definitively established from the available data. Variations in sample sizes, community settings, and participant demographics represent plausible explanatory factors. The higher distances observed in the National Veterans Wheelchair Games (NVWG) could reflect the more active lifestyle characteristics typical of competitive athletes, as competitive sport participation has been associated with higher physical activity levels in other disabled populations. However, other unmeasured factors such as differences in measurement protocols, environmental contexts, or participant motivation may have also influenced these findings. Similarly, while previous research has documented associations between employment status and greater wheeled distances***

***[14,15], the extent to which employment or other socioeconomic factors explain cross-study differences remains uncertain. Geographic and cultural differences between studies may also contribute, but identifying the specific mechanisms linking countries of origin to mobility patterns would require further investigation. Future research should prioritize standardized measurement protocols and more comprehensive demographic characterization to enable more definitive conclusions about factors influencing daily mobility patterns in wheelchair users."***

- More explicitly discuss whether the observed longer bouts in your study could reflect true behavioral or contextual differences, and not just methodological differences.

  **<u>Authors' response:</u>**

  **We appreciate this comment and have extended our discussion on this point as follows:**

  ***"Our analysis of bout lengths revealed some differences compared to previous research. We found longer mean bout lengths than previously reported [12]. While these differences can be partially attributed to variations in mobility bout definitions across studies, they may also reflect genuine behavioral and contextual differences in mobility patterns.***

  ***Methodologically, our study defined a bout as a period of wheelchair propulsion capturing intentional transitions between activities with pauses less than one minute (beginning when wheelchair speed exceeded 0.1 m/s with at least one complete wheel revolution), whereas previous research used different criteria: bouts lasting at least 5 seconds, with speeds ≥ 0.12 m/s, ending when less than 0.76 m were wheeled within 15 seconds [12]. These different definitions likely account for the observed variations, although they might also reflect genuine differences in mobility patterns influenced by community environments.***

  ***However, the longer bout lengths in our study may also indicate true differences in mobility behavior and environmental context. Our participants' community environments may have promoted longer, more sustained periods of wheelchair propulsion compared to previous studies. Factors such as greater distances between destinations, more accessible infrastructure encouraging continuous movement, or different activity patterns within the community could contribute to these extended mobility bouts. Additionally, our participants may have engaged in different types of activities or had mobility goals that naturally resulted in longer propulsion periods.***

  ***We defined three distinct categories of movement bouts based on duration, with short bouts (< 215s) comprising nearly 94% of weekly activity. The high standard deviations in our bout characteristics also suggest considerable variability in individual mobility, which may reflect both personal preferences and diverse environmental demands within the community setting."***

- The high intra-individual variability is an important finding, but its implications are only lightly addressed. Expand on how such variability could influence rehabilitation planning, device design, or health monitoring strategies.
  **<u>Authors' response:</u>**
  **<u>We have expanded on this point:</u>**
  ***"...This high intra-individual variability has several important clinical and technological implications. For rehabilitation planning, it suggests that mobility assessments based on single-day or short-term observations may not accurately represent an individual's typical patterns, potentially leading to suboptimal therapeutic targets. Clinicians should consider incorporating week-long monitoring to establish realistic baseline mobility goals and track meaningful changes over time. For device design, this variability indicates that 'one-size-fits-all' wheelchair configurations may be inadequate; incorporating adaptive or customizable features that accommodate daily fluctuations in mobility demands could improve user satisfaction and reduce overuse injuries. In health monitoring strategies, understanding an individual's typical variability range allows for better detection of clinically significant changes that warrant intervention, distinguishing between normal day-to-day fluctuations and concerning mobility declines..."***

- The limitations are clearly acknowledged, which is good. However, the potential impact of these limitations on specific results or interpretations is not fully articulated. Be more explicit about how these limitations might affect the validity or generalizability of the findings.
  **<u>Authors' response:</u> Great comment. The impact has been expanded as follows:**

  ***"...These limitations may have resulted in underestimation of true mobility patterns and biomechanical loading in some participants, potentially affecting the accuracy of our proposed mobility profiles. The missing wheelchair data for participants using multiple chairs likely led to incomplete capture of daily mobility demands, which could influence the validity of mobility-health relationships derived from our data. Additionally, the inability to account for different surface types means our biomechanical interpretations may not fully represent the loading experienced during real-world mobility, limiting the clinical applicability of these findings for predicting shoulder pathology risk..."***

**<u>Conclusion</u>**

- While the conclusion notes variability and the need for personalized assessment, it doesn't fully explain how this could be translated into interventions or clinical practice. Add a line or two about how your metrics could inform practical applications, such as rehabilitation protocols, assistive device design, or long-term health monitoring.
- Also, tie your findings back to larger goals: autonomy, inclusion, public health, etc.

**<u>Authors' response:</u> Thank you for this important guidance. We have attempted to address these points in the edited conclusion below.**

***"These individualized mobility metrics have direct translational potential for clinical practice and intervention development. For instance, users demonstrating high frequencies of starts/stops or steep slope navigation could be prioritized for targeted strength training programs or fitted with power-assist technologies to reduce upper extremity loading. Rehabilitation protocols could be personalized based on mobility profiles, for example, those with extended continuous propulsion might benefit from endurance conditioning, while users with frequent directional changes could receive specialized training in efficient maneuvering techniques. Additionally, these objective data could inform evidence-based wheelchair prescription by matching device specifications to individual usage patterns and provide quantitative baselines for monitoring functional changes during routine clinical follow-ups. From a broader perspective, this work supports fundamental goals of autonomy and community inclusion by developing precise, individualized approaches to prevent secondary health conditions that could otherwise limit full participation in employment, recreation, and social activities. By reducing the burden of preventable shoulder injuries, this research contributes to public health objectives of maintaining functional independence, reducing healthcare costs, and enabling aging in place for manual wheelchair users."***

---

The reference list has been updated with the corrected reference 10 and four additional references (29, 30, 34 and 39) have been incorporated. These additions appear to strengthen the literature base with relevant research on clustering optimization methods and IMU-based ergonomic assessments for wheelchair users.

**Reference 17 (Updated):** de Vries WHK, van der Slikke RMA, van Dijk MP, Arnet U. Real-Life Wheelchair Mobility Metrics from IMUs. Sensors. 2023;23: 7174. doi:10.3390/s23167174

**New Reference 29 (Added):** Madgwick S. Fusion: Sensor fusion library for Inertial Measurement Units (IMUs), version 1.2.8 [Internet]. GitHub; 2025 Jan 30 [cited 2025 Aug 13]. Available from: https://github.com/xioTechnologies/Fusion

**New Reference 30 (Added):** Kuipers JB. Quaternions and Rotation Sequences: A Primer with Applications to Orbits, Aerospace, and Virtual Reality. . Princeton, NJ: Princeton, NJ: Princeton University Press.; 2007.

**New Reference 34 (Added):** Umargono E, Suseno JE, Vincensius Gunawan SK. K-Means Clustering Optimization Using the Elbow Method and Early Centroid Determination Based on Mean and Median Formula. Proceedings of the 2nd International Seminar on Science and Technology (ISSTEC 2019). Paris, France: Atlantis Press; 2020. doi:10.2991/assehr.k.201010.019

**New Reference 39 (Added):** Jahanian O, Van Straaten MG, Goodwin BM, Cain SM, Lennon RJ, Barlow JD, et al. Inertial Measurement Unit–Derived Ergonomic Metrics for Assessing Arm Use in Manual Wheelchair Users With Spinal Cord Injury: A Preliminary Report. Top Spinal Cord Inj Rehabil. 2021;27: 12–25. doi:10.46292/sci20-00059
